# Superiority of albumin–globulin ratio over albumin to predict mortality in patients undergoing peritoneal dialysis

**DOI:** 10.1038/s41598-020-73629-5

**Published:** 2020-11-13

**Authors:** Chun-Chieh Tsai, Yao-Peng Hsieh, Shr-Mei Tsai, Chew-Teng Kor, Ping-Fang Chiu

**Affiliations:** 1 Division of Nephrology, Department of Internal Medicine, Changhua Christian Hospital, 135 Nanxiao Street, Changhua City, 500 Taiwan, ROC; 2 School of Medicine, Kaohsiung Medical University, Kaohsiung, Taiwan; 3 School of Medicine, Chung Shan Medical University, Taichung, Taiwan; 4 Department of Nursing, Changhua Christian Hospital, Changhua, Taiwan; 5 Department of Recreation and Holistic Wellness, MingDao University, Changhua, Taiwan

**Keywords:** Nephrology, Risk factors

## Abstract

There is increasing evidence showing that albumin–globulin ratio (AGR) can predict the survival of patients in many types of malignancies. However, no study was done to explore the value of AGR in peritoneal dialysis (PD) patients. A total of 554 incident patients undergoing PD from January 2001 through July 2016 were enrolled for this retrospective observational study. The outcomes of interest were all-cause mortality and cardiovascular disease (CVD) mortality. Baseline patient’s socio-demographic data, pharmacotherapy, comorbidities, laboratory and PD-related parameters were collected and used in the multivariate Cox models. The predictive value of AGR on mortality risk was compared with other markers using area under the receiver operating characteristic curve (AUC) analysis. Among the study participants, there were 265 (47.83%) men and the mean follow-up time was 3.87 ± 3.15 years. Univariate Cox analysis showed that low AGR was significantly associated with worse outcomes in terms of all-cause and CVD mortality and it remained an independent predictor in the multivariate models. The fully adjusted hazard ratios for the low AGR group versus high AGR group were 2.12 (95% CI 1.34–3.35, p = 0.001) and 2.58 (95% CI 1.42–4.7, p = 0.002) for all-cause and CVD mortality, respectively. The predictive ability of AGR for mortality risk was superior to that of other biomarkers based on AUC calculations. In conclusion, low AGR was independently associated with higher all-cause and CVD mortality risks in patients undergoing PD.

## Introduction

Although the treatment strategies to improve survival in chronic kidney disease (CKD) patients have been developed in the past few decades, the prognosis still remains poor. Recently, several clinical and biochemical biomarkers, including serum level of gut microbiota-dependent trimethylamine N-oxide, aldosterone and urine neutrophil gelatinase-associated lipocalin concentration, have been identified as crucial prognostic factors for cardiovascular events or mortality in patients with CKD in previous reports^1–3^. However, these factors may be either inaccessible in clinical practice, costly or unstable to reproduce. Thus, it is urgent to identify promising prognostic markers which are clinically available, accurate and efficient for the purpose of risk stratification. Clinical management guided by this novel predictor could improve their long-term survival.

Albumin and globulin, the two main constituents in the serum protein, have been reported to be implicated in the nutrition and systemic inflammation. Hypoalbuminemia is not only an index of malnutrition but also reflects the status of chronic inflammation^4^. On the other hand, high globulin could be the results of chronic inflammation and the responses to various proinflammatory cytokines^5^. High albumin level or low globulin level is associated with better survival in patients with breast cancer, ovarian cancer or colorectal cancer^6–8^. To date, interestingly, there is increasing evidence showing that albumin–globulin ratio (AGR) can predict the survival of patients in many types of malignancies^9, 10^. More recently, Zhou et al. reported AGR to be an independent factor for predicting overall survival in patient with lung cancer^11^.

CKD is a clinical condition characterized by the propensity of poor nutrition and high levels of inflammation. Hypoalbuminemia is a significant risk factor for morbidity and mortality in these patients^12^. As expected, systemic inflammation indeed independently predicted patient survival in both prevalent and incident patients undergoing peritoneal dialysis (PD)^13^. However, no study was done to explore the value of AGR in PD patients. Thus, this study was conducted to assess the effect of AGR on predicting patient survival in patients undergoing PD. Moreover, we also compared the prognostic value between AGR and other clinical indexes.

## Results

### Patients’ baseline characteristics

From January 1st 2001 to July 31st 2016, 554 patients were eligible for this study with 47.83% male and the mean follow-up time was 3.87 ± 3.15 years. Table 1 showed the baseline characteristics of these patients by AGR stratification into the high AGR and the low AGR groups. The age at the study enrollment was older in the low AGR group than in the high AGR group (56.71 ± 15.56 vs. 47.95 ± 14.07, p < 0.001). A higher proportion of patients in the low AGR group had type 2 DM and cardiovascular disease than the high AGR group. About the use of drugs, more patients in the high AGR group were prescribed vitamin D supplement. In addition, there was a marked difference in the PD-related parameters between the two AGR groups.

**Table 1:** Baseline characteristics of the study population by the AGR groups.

	AGR ≥ 1.2	AGR < 1.2	P-value
Number of patients	240	314	–
Sex, men	113 (47.08%)	152 (48.41%)	0.757
Age (years)	47.95 ± 14.07	56.71 ± 15.56	< 0.001*
Body mass index (kg/m^2^)	23.36 ± 3.86	23.5 ± 3.9	0.679
**Smoker**			
Never	195 (81.25%)	261 (83.12%)	0.646
Current	7 (2.92%)	5 (1.59%)	0.443
Former	38 (15.83%)	48 (15.29%)	0.954
**Medications**			
ACE inhibitor/ARB	142 (59.17%)	201 (64.01%)	0.244
Erythropoiesis-stimulating agents	227 (94.58%)	290 (92.36%)	0.298
Vitamin D	84 (35%)	70 (22.29%)	0.001*
Calcium channel blocker	163 (67.92%)	226 (71.97%)	0.301
**Comorbid conditions**			
Hypertension	203 (84.58%)	268 (85.35%)	0.802
Diabetes mellitus	46 (19.17%)	140 (44.59%)	< 0.001*
Cardiovascular disease	51 (21.25%)	149 (47.45%)	< 0.001*
Hyperlipidemia	46 (19.17%)	96 (30.57%)	0.002*
**Status prior to PD**			
Predialysis CKD	204 (85%)	258 (82.17%)	0.439
Hemodialysis	36 (15%)	56 (17.83%)	0.439
**Causes of CKD**			
chronic glomerulonephritis	104 (43.33%)	73 (23.25%)	< 0.001*
hypertension	44 (18.33%)	53 (16.88%)	0.739
Diabetes mellitus	34 (14.17%)	121 (38.54%)	< 0.001*
Others	58 (24.17%)	67 (21.34%)	0.492
**Laboratory data**			
Albumin to globulin ratio	1.4 (1.3, 1.6)	1.0 (0.9, 1.1)	< 0.001*
Serum albumin (g/dL)	3.8 (3.4, 4.1)	3.0 (2.6, 3.3)	< 0.001*
Total protein (g/dL)	6.4 (5.8, 6.9)	6.1 (5.4, 6.7)	< 0.001*
Calcium (mg/dL)	8.4 (8, 8.9)	8.3 (7.9, 8.8)	0.103
Phosphorus (mg/dL)	5.7 (5.0, 6.35)	5.3 (4.4, 6.2)	< 0.001*
Cholesterol (mg/dL)	185 (158, 213)	184 (153, 225)	0.676
Triglyceride (mg/dL)	115 (86, 157)	123 (91, 172)	0.145
Creatinine (mg/dL)	10.27 (8.61, 12.62)	9 (7.51, 10.66)	< 0.001*
Ferritin (ng/mL)	259.3 (131.1, 438)	262.4 (131.3, 546.4)	0.259
GPT (U/L)	15 (12, 22)	17 (12, 24)	0.11
Hemoglobin (g/dL)	8.6 (7.6, 9.4)	8.8 (8, 9.5)	0.015*
Intact PTH (pg/mL)	381 (209.6, 606)	307.7 (184, 485.08)	0.004*
WBC count (×10^3^/μL)	6.7 (5.35, 8)	7.49 (6.2, 9.2)	< 0.001*
**PD-related parameters**			
D/P creatinine at 4 h	0.66 (0.59, 0.73)	0.69 (0.6, 0.78)	0.002*
24-h urine volume (L)	1.05 (0.63, 1.41)	0.73 (0.42, 1.15)	< 0.001*
Weekly total Kt/V urea	2.12 (1.84, 2.4)	1.95 (1.64, 2.26)	< 0.001*
nPNA (g/kg/day)	1.11 (0.88, 1.29)	1.01 (0.8, 1.17)	< 0.001*
Residual renal function (mL/min/1.73 m^2^)	3.01 (2.33, 3.98)	2.89 (1.87, 3.82)	0.05*

Values are expressed as mean ± SD, median and interquartile range, or number (percentage).

AGR, albumin–globulin ratio; ACE inhibitor, angiotensin-converting enzyme inhibitor; ARB, angiotensin II receptor blocker; GPT, glutamic-pyruvic transaminase; WBC, white blood cell; PTH, parathyroid hormone; D/P creatinine, dialysate-to-plasma creatinine ratio; nPNA, normalized protein nitrogen appearance; PD, peritoneal dialysis.

*p-value < 0.05.

### Association of AGR with all-cause and CVD-related mortality

**Figure 1:**
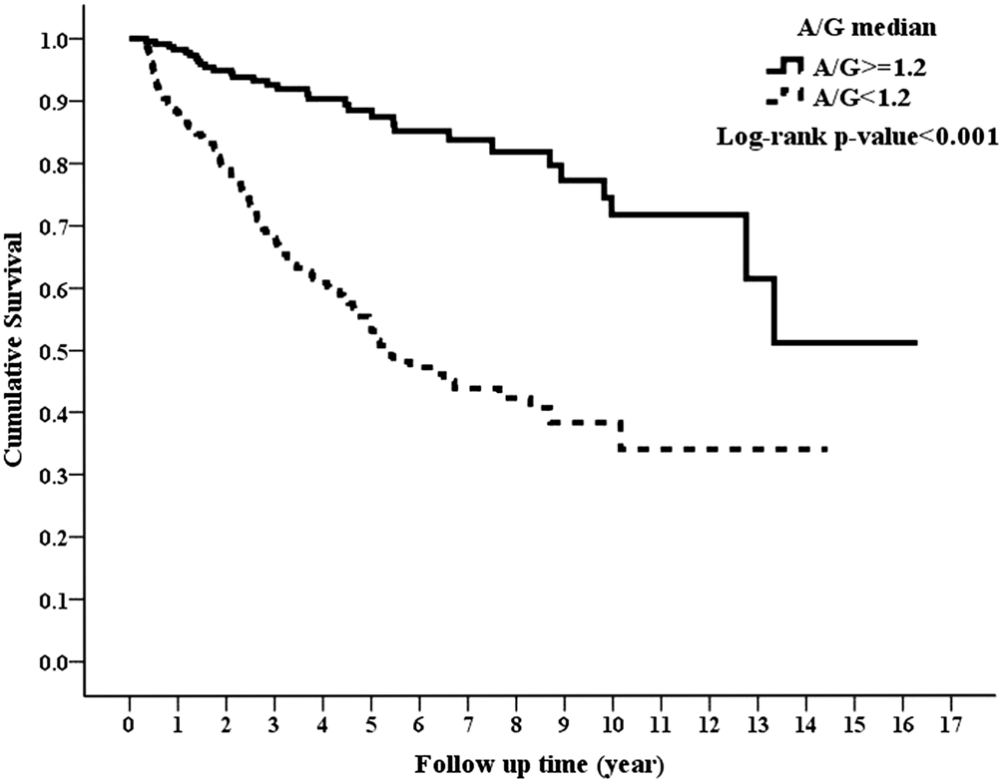
Kaplan–Meier curve of overall patient survival according to the AGR groups (log-rank test, p < 0.001).

**Figure 2:**
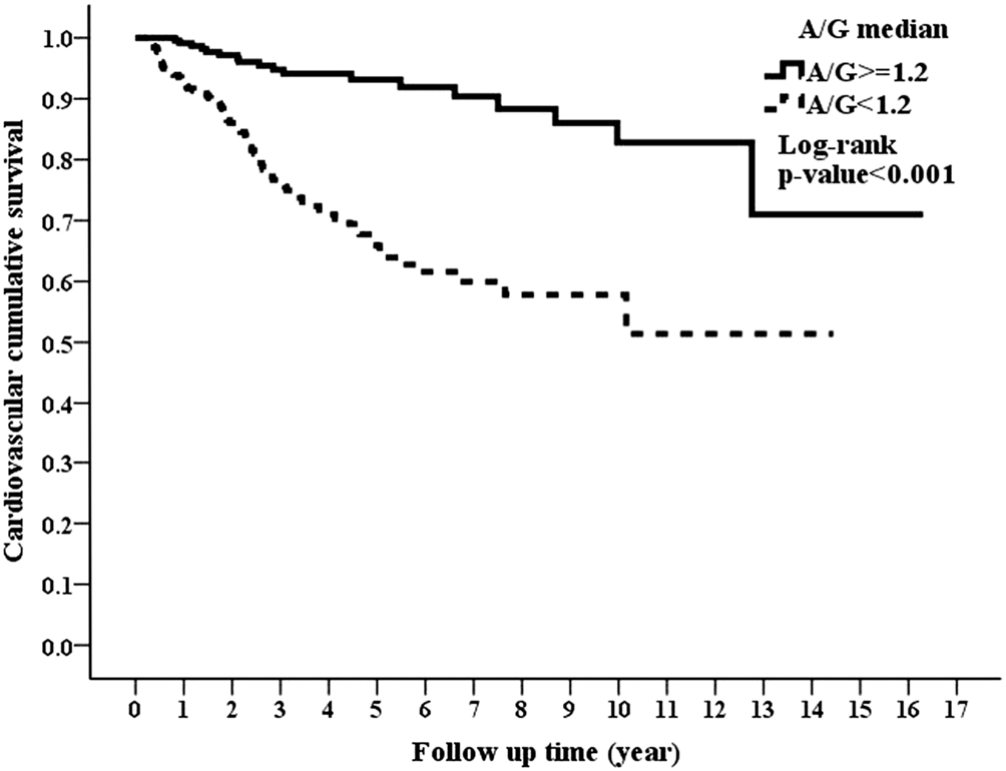
Kaplan–Meier curve of cardiovascular cumulative survival according to the AGR groups (log-rank test, p < 0.001).

During the follow-up period of nearly 3.87 years, there were 31 (12.92%) and 120 (38.22%) patients who died in the high and low AGR groups, respectively (p < 0.001). CVD, which was the leading cause of death in our study cohort, accounted for 18 (7.5%) and 77 (24.5%) deaths in the high and low AGR groups, respectively (p < 0.001). Kaplan–Meier survival plots unveiled that the high AGR group had better overall and CVD cumulative survival than the low AGR group (Figs. 1 and 2; p < 0.001, p < 0.001, respectively). In the unadjusted Cox model, low AGR group was associated with a higher all-cause mortality risk of HR 4.16 (95% CI 2.79–6.2) and CVD mortality risk of HR 4.52 (95% CI 2.69–7.58) in comparison with high AGR group. The significant differences in all-cause and CVD mortality continued from model 1 to model 5, where the fully adjusted HRs of low AGR group versus high AGR group were 2.12 (95% CI 1.34–3.35, p = 0.001) and 2.58 (95% CI 1.42–4.7, p = 0.002) for all-cause and CVD mortality, respectively (Table 2).

**Table 2:** Univariate and multivariate Cox regression models of all-cause and cardiovascular mortality for AGR groups (all patients = 554).

	All-cause mortality		CVD mortality	
	Hazard ratio (95% CI)	p-value	Hazard ratio (95% CI)	p-value
**(A) AGR median ( AGR < 1.2 vs. AGR ≥ 1.2)**				
Univariate model	4.16 (2.79, 6.2)	< 0.001	4.52 (2.69, 7.58)	< 0.001
Model 1	2.46 (1.63, 3.73)	< 0.001	2.76 (1.62, 4.72)	< 0.001
Model 2	2.64 (1.74, 4.02)	< 0.001	2.96 (1.73, 5.09)	< 0.001
Model 3	2.44 (1.59, 3.73)	< 0.001	2.69 (1.56, 4.64)	< 0.001
Model 4	2.32 (1.51, 3.58)	< 0.001	2.51 (1.44, 4.39)	0.001
Model 5	2.12 (1.34, 3.35)	0.001	2.58 (1.42, 4.70)	0.002
**(B) Sensitivity tests**				
(i) AGR as a continuous variable (per 1-unit decrease)	2.89 (1.79, 4.64)	< 0.001	3.27 (1.82, 5.88)	< 0.001
(ii) Optimal AGR by ROC analysis	2.13 (1.43, 3.17)	< 0.001	2.12 (1.28, 3.51)	0.004
(iii) AGR tertiles				
Third tertile	1		1	
Second tertile	1.68 (0.99, 2.84)	0.052	2.32 (1.2, 4.5)	0.012
First tertile	2.57 (1.57, 4.21)	< 0.001	2.84 (1.49, 5.41)	0.001
P for trend	–	< 0.001	–	0.002
(iv) AGR median adjusted for propensity score	1.74 (1.13, 2.66)	0.012	1.81 (1.04, 3.14)	0.037

Model 1: AGR median, age, sex, BMI and smoking status.

Model 2: Model 1 plus medications.

Model 3: Model 2 plus comorbidities.

Model 4: Model 3 plus peritoneal dialysis-related parameters.

Model 5: model 4 plus laboratory data.

AGR, albumin–globulin ratio.

### Sensitivity analysis

AGR remained a significant predictor for overall and CVD mortality regardless of whether analyses were performed with AGR as a continuous covariate, AGR tertiles, optimal AGR value by ROC analysis, or adjustment for propensity score (Table 2).

### Predictive value of AGR

We further compared the prognostic ability of AGR with that of other biochemical markers, including albumin, hemoglobin, ferritin, total protein and WBC counts for predicting mortality risk by AUC calculation in ROC analysis. As shown in Table 3, AGR had the highest predictive capacity over other biochemical data in predicting mortality risk of all-cause and CVD mortality within 1-year, 3-year, 5-year and the whole study period. While we added these variables separately to variables in model 4 to compared the AUC of them, AGR also showed its superiority in predicting mortality risk, except for the overall CVD mortality. Similarly, the model improvement using integrated discrimination improvement (IDI) was 4.0% and 5.1% higher in predicting overall all-cause and CVD mortality while adding AGR (Table 4). Using IDI, the addition of AGR was associated with the highest model improvement in predicting all-cause and CVD mortality within 1-year, 3-year, 5-year and the whole period compared with other covariates.

**Table 3:** AUC using ROC curve analysis to predict all-cause and cardiovascular mortality by various parameters.

	All-cause mortality				Cardiovascular mortality			
	1-year	3-year	5-year	Overall	1-year	3-year	5-year	Overall
**AUC for each variable**								
AGR	**0.788 (0.72, 0.74)**	**0.755 (0.70, 0.77)**	**0.789 (0.73, 0.76)**	**0.638 (0.35, 0.71)**	**0.790 (0.69, 0.89)**	**0.746 (0.67, 0.82)**	**0.801 (0.73, 0.87)**	**0.757 (0.66, 0.85)**
Albumin	0.734 (0.67, 0.68)	0.690 (0.63, 0.70)	0.720 (0.65, 0.76)	0.508 (0.17, 0.10)	0.698 (0.61, 0.78)	0.692 (0.61, 0.78)	0.726 (0.64, 0.81)	0.730 (0.61, 0.85)
Hemoglobin	0.537 (0.45, 0.52)	0.556 (0.49, 0.58)	0.556 (0.50, 0.58)	0.542 (0.38, 0.36)	0.613 (0.50, 0.73)	0.587 (0.51, 0.66)	0.588 (0.52, 0.66)	0.576 (0.49, 0.66)
Ferritin	0.700 (0.61, 0.79)	0.607 (0.54, 0.67)	0.603 (0.54, 0.67)	0.540 (0.43, 0.65)	0.612 (0.50, 0.72)	0.557 (0.48, 0.64)	0.542 (0.46, 0.62)	0.529 (0.43, 0.63)
Total protein	0.526 (0.43, 0.62)	0.526 (0.45, 0.6)	0.553 (0.48, 0.62)	0.540 (0.41, 0.67)	0.452 (0.33, 0.57)	0.522 (0.44, 0.60)	0.548 (0.47, 0.63)	0.561 (0.48, 0.64)
White blood cell count	0.732 (0.65, 0.81)	0.636 (0.57, 0.70)	0.634 (0.57, 0.69)	0.618 (0.54, 0.70)	0.736 (0.62, 0.85)	0.629 (0.55, 0.71)	0.644 (0.56, 0.72)	0.630 (0.54, 0.72)
**AUC for variables in model 4 plus the following variable**								
AGR	**0.916 (0.88, 0.95)**	**0.913 (0.88, 0.95)**	**0.919 (0.87, 0.96)**	**0.911 (0.84, 0.99)**	**0.905 (0.85, 0.96)**	**0.931 (0.88, 0.98)**	**0.956 (0.91, 1)**	0.93 (0.83, 1.03)
Albumin	0.908 (0.87, 0.94)	0.906 (0.87, 0.94)	0.911 (0.86, 0.96)	0.896 (0.82, 0.98)	0.899 (0.84, 0.96)	0.924 (0.88, 0.97)	0.941 (0.89, 0.99)	**0.932 (0.84, 1.03)**
Hemoglobin	0.895 (0.85, 0.94)	0.897 (0.86, 0.93)	0.9 (0.86, 0.94)	0.886 (0.81, 0.96)	0.887 (0.82, 0.95)	0.914 (0.86, 0.97)	0.927 (0.87, 0.98)	0.924 (0.82, 1.03)
Ferritin	0.898 (0.86, 0.94)	0.899 (0.86, 0.94)	0.902 (0.86, 0.95)	0.884 (0.81, 0.96)	0.881 (0.81, 0.95)	0.917 (0.87, 0.97)	0.93 (0.88, 0.98)	0.923 (0.82, 1.03)
Total protein	0.89 (0.84, 0.93)	0.896 (0.86, 0.93)	0.896 (0.85, 0.94)	0.887 (0.82, 0.96)	0.883 (0.81, 0.95)	0.913 (0.86, 0.96)	0.926 (0.87, 0.98)	0.92 (0.81, 1.03)
White blood cell count	0.898 (0.86, 0.94)	0.897 (0.86, 0.93)	0.901 (0.86, 0.95)	0.882 (0.81, 0.95)	0.894 (0.82, 0.97)	0.919 (0.87, 0.97)	0.934 (0.88, 0.99)	0.916 (0.81, 1.02)

Data are presented as AUC (95% confidence interval).

Variables in model 4 included age, sex, body mass index, smoking status, medications usage, comorbid conditions and peritoneal dialysis-related parameters.

Figures in **bold** type indicated the largest AUC value in each of specified categories.

AUC, area under the curve; ROC, receiver operating characteristic; AGR, albumin–globulin ratio.

**Table 4:** Test of discriminatory value to evaluate predictive value of AGR in comparison to other laboratory variables.

IDI (%) with addition of each variable	All-cause mortality				CVD mortality			
	1-year	3-year	5-year	overall	1-year	3-year	5-year	Overall
AGR	3.0 (0.2, 7.6)*	1.5 (− 0.1, 4.3)	2.7 (0.4, 6.5)*	4.0 (0.6, 8.1)*	2.9 (0, 7.2)*	0.7 (− 0.9, 3.5)	2.4 (0, 6.3)*	5.1 (− 1.4, 13.5)
Albumin	0.5 (− 0.4, 2)	0 (− 0.5, 1.3)	0.6 (− 0.5, 2.9)	1.0 (− 0.8, 4.0)	0 (− 0.6, 0.8)	− 0.2 (− 0.9, 1.2)	0.5 (− 0.3, 3.0)	1.4 (− 2.1, 6.3)
Hemoglobin	0 (− 0.3, 0.6)	0 (− 0.6, 0.9)	0 (− 0.4, 0.6)	0 (− 0.9, 3.7)	0 (− 0.6, 1.2)	0 (− 0.7, 1.2)	0 (− 0.3, 0.8)	0 (− 1.6, 3.9)
Ferritin	0 (− 0.6, 2.1)	0 (− 0.3, 0.8)	0 (− 0.2, 0.5)	0 (− 0.6, 1.1)	0 (− 0.4, 1.1)	0 (− 0.4, 0.8)	0 (− 0.4, 0.9)	0 (− 1.9, 2)
Total protein	0.7 (− 0.5, 3.1)	0.7 (− 0.5, 2.7)	0 (− 0.6, 1.3)	0.1 (− 0.8, 1.4)	1.2 (− 0.4, 4.5)	0.6 (− 0.7, 2.9)	− 0.1 (− 0.7, 1.5)	− 0.7 (− 3.0, 1.2)
WBC	0 (− 1.5, 2.4)	0 (− 0.4, 0.8)	0 (− 0.7, 1.2)	0 (− 0.9, 1.2)	0 (− 1.6, 3.7)	0 (− 0.3, 1.3)	0 (− 0.6, 1.3)	0 (− 1.7, 2.4)

Data was presented in percentage (%).

AGR, albumin–globulin ratio; IDI, integrated discrimination improvement; WBC, white blood cell.

*P-value < 0.05.

### Correlation between AGR and clinical parameters

We also examined the strength of association and correlation of AGR with laboratory and PD- related data by multiple linear regression analyses and Pearson correlation test (Table 5). AGR was positively correlated with nutritional maker (serum creatinine), and negatively correlated with inflammatory markers (WBC counts and ferritin).

**Table 5:** Linear regression coefficients and Pearson correlation of AGR with various laboratory and peritoneal dialysis-related data.

	Coefficients (95% CI)	Pearson correlation	p-value
Calcium	0.055 (0.02,0.09)	0.140	0.001*
Creatinine	0.028 (0.02,0.03)	0.304	< 0.001*
Hemoglobin	− 0.018 (− 0.04,0.00)	− 0.078	0.063
WBC counts	− 0.022 (− 0.03,− 0.01)	− 0.184	< 0.001*
D/P creatinine at 4 h	− 0.243 (− 0.42,− 0.06)	− 0.103	0.009*
24-h urine output	0.102 (0.06,0.14)	0.204	< 0.001*
iPTH (per 100 unit increment)	− 0.009 (− 0.02,0.00)	− 0.097	0.020*
Ferritin (per 100 unit increment)	− 0.005 (− 0.01,0.00)	− 0.077	0.047*
Triglyceride (per 100 unit increment)	− 0.024 (− 0.05,0.00)	− 0.079	0.043*

AGR, albumin–globulin ratio; WBC, white blood cell; PTH, parathyroid hormone; D/P creatinine, dialysate-to-plasma creatinine ratio.

## Discussion

In this study, we tested the hypothesis that baseline AGR is of predictive value for mortality risk among PD patients and found that low AGR was associated with poor patient survival in a cohort of 554 incident PD patients over a mean follow-up period of 3.87 years. The results of multivariate analysis showed that this association was independent of many clinically relevant confounding factors. Consistent results in the sensitivity tests increased the robustness of our findings. Moreover, the predictive value of AGR for mortality was superior to that of other laboratory indexes, including albumin.

Albumin, the most abundant serum protein, is usually used to reflect the nutritional status and can function to balance blood PH level and maintain intravascular volume. The serum albumin levels were inversely associated with major cardiovascular events^14–16^. A progressive consumption of protein and/or energy is commonly observed in CKD patients. Apart from reduced nutrition intake, the causes of hypoalbuminemia in CKD/ESRD patients include hypercatabolism, metabolic acidosis, reduced physical activity, reduced anabolism, chronic inflammation, oxidative stress, comorbidities, life style and dialysis treatment^17^. The term “Protein-Energy Wasting (PEW)” has been used to describe this entity of protein/energy wasting, malnutrition and inflammation. Protein-energy malnutrition is a crucial constituent of PEW and the coexistence of inflammation and muscle wasting is the hallmark of CKD- related PEW, distinguishing itself from other forms of poor nutrition. PEW is highly prevalent in CKD patients, whose prevalence varied a lot depending on the severity of CKD and study population, and is closely correlated with adverse clinical impact and high risk for mortality^18–21^. The suggested methods for defining PEW include nutritional intake, body mass and composition, subjective global assessment, malnutrition-inflammation score and laboratory measurements^22^. Of the numerous laboratory tests, serum albumin is one of the most used index and strongly predicts mortality. Furthermore, the association of the change in nutritional status with overall mortality was claimed by Kwon et al. who demonstrated that the mortality rate in patients whose nutritional status was poor at the dialysis commencement but improved after 12 months was comparable to those patients having good nutritional status at both time points^23^. Thus, proper interventions to improve nutritional status could improve patient survival in malnourished patients.

In addition to be a nutritional index, in vitro experimental studies demonstrated the antiplatelet aggregation effect of albumin, which was possibly attributed to the negatively charged entity^24, 25^. Other studies found the antithrombogenicity effects of albumin through the inhibition of thromboxane A2 synthesis via increasing the formation of prostaglandin D2 and sequestering the liberated arachidonic acid^26, 27^. Interesting, a study of patients undergoing peritoneal dialysis showed that reduced platelet aggregation was achieved after receiving albumin to correct hypoalbuminemia^28^. Thus, albumin is emerging to be inversely related to atherogenesis and thrombosis, which lead to high mortality risk.

Globulins, the main constituents of non-albumin proteins in the serum, can carry sex hormones and fatty acid, and play a key role in the maintenance of osmotic pressure, immunity and inflammation^29^. Thus, higher levels of serum globulins, resulting from the accumulation of immunoglobulins and acute phase proteins, are reflective of a more severe degree of inflammatory response. In the present study, significantly negative associations between the AGR levels and WBC and ferritin were observed, consistent with the inflammatory marker of globulins. Inflammation has been recognized as a well-established comorbid condition in CKD and especially in dialysis patients over the past decades. The contributing factors of chronic inflammation in CKD include increased production and decreased clearance of pro-inflammatory cytokines, oxidative stress, acidosis, altered metabolism of adipose tissue, intestinal dysbiosis and current infection^30^. Persistent inflammation plays a pathophysiological role in the mortality risk. Several inflammatory markers, including C-reactive protein (CRP), WBC, IL-1, IL-6 and TNF-alpha, have ever been reported to be predictive of all-cause and/or cardiovascular mortality in CKD patients^30^. As mentioned above, persistent inflammation also contributes to the development of PEW. The mechanistic explanations of inflammation-induced PEW involve anorexia, depression, increased resting energy expenditure and suppression of anabolic hormones^30, 31^. A variety of interventions that have been done to mitigate inflammation in CKD include lifestyle modification, pharmacologic therapy, and optimization of dialysis. However, further large-scale investigations are required to corroborate their effects.

Inflammation and nutritional status are mutually and negatively influenced while the former can be represented by globulin and the later by albumin. As serum albumin is largely influenced by inflammation, changes in hepatic synthesis, catabolism and hydration status, the use of AGR, an integrate of albumin and globulin, would be less affected by the factors above and might provide a more stable and reliable biochemical index than either index alone.

As we know, low serum albumin was demonstrated to be an independent predictor of poor survival from the considerable amount of previous research. In contrast, less interest has been given to the prognostic impact of AGR on patient survival. Most of the investigations on AGR mainly focused on patients with malignancies over the last decade. Previous studies demonstrated that low AGR was predictive for poor survival in many types of malignancies, including breast cancer, colorectal cancer, lung cancer and nasopharyngeal cancer^9–11, 29, 32^. The work on the role of AGR in the non-cancerous patients is limited so far. Beamer et al. reported low AGR was significantly linked with a higher risk of subsequent vascular events in patients with stroke and in high-risk stroke participants^33^. Later, a study of patients with non-ST elevation myocardial infarction was carried out by Azab et al. showing that AGR is a significant predictor of long-term mortality after adjusting for 20 confounding factors and is superior to albumin as a predictor of mortality^34^. To the best of our knowledge, the present study was the first work showing the prognostic role of AGR in PD patients. A low AGR may indicate the presence of low albumin, high globulin, or both. Given that malnutrition is potentially reversible in nature, early identification of high-risk patients for malnutrition with appropriate nutritional intervention may improve the quality of life and reduce mortality rate. Moreover, novel therapeutic strategies to reduce systemic inflammation might represent a promising way to mitigate mortality risk in that high-risk population. Thus, more medical attention should be paid to those patients with a low AGR with the aim of improving nutritional status or reducing inflammation.

There were also some limitations for this study. First, the retrospective and observational nature of this investigation also limits its rigorousness and persuasiveness. Single-center design may also prevent our results from being generalized to PD patients worldwide. Second, we did not have the measurements of other specific inflammatory proteins, such as cytokine and C-reactive protein. Instead, the adjusted variables included WBC counts and ferritin, which are also considered inflammatory markers. Third, we only took one measurement of AGR at baseline for evaluation. The influence of time-dependent variation in AGR on clinical outcomes cannot be addressed in this study. Despite these limitations, our study represented the first one investigating the predictive role of AGR in the risk of mortality amongst PD patients and the association was independent of many crucial clinical parameters. AGR is superior to albumin in predicting the risk of all-cause mortality and CVD mortality.

In conclusion, AGR, the ratio of the two significant and inversely related predictors, played an important role in predicting the risk for mortality among PD patients. Patients with low AGR were prone to have a higher risk of mortality. Furthermore, AGR might be considered as a novel biomarker for defining PEW in patients undergoing PD. In addition, in combination with other significant predictors, AGR could assist clinicians in providing individualized health care based on risk stratification. Further large and prospective studies of multicenter design are required to corroborate our findings and the mechanistic explanations underlying our conclusions at the molecular level also need to be elucidated.

## Materials and methods

### Participants and measurements

This study was carried out to retrospectively investigate the prognostic significance of albumin–globulin ratio (AGR) in the risk for mortality among PD patients. Incident patients who underwent PD for end stage renal disease in Changhua Christian Hospital peritoneal dialysis unit between January 2001 and July 2016 were selected into this study. Exclusion criteria were as the following: patients who were young than 18 years of age and those who had undergone PD for less than 3 months. Therefore, a group of 554 patients were eligible for the final study analysis. All the patients were followed from the date of study entry, defined as the date of initiating PD, till patient death or 31 July 2017.

Patients’ baseline characteristics were collected from the established computerized database. Routine demographic characteristics were recorded at study enrollment and included gender, age, body mass index, smoking status, the underlying etiology of CKD and the pre-dialysis status, which was categorized as pre-dialysis CKD, failed kidney transplant and transfer from hemodialysis. PD-related measurements included normalized protein nitrogen appearance (nPNA), residual renal function (the average of 24-h urine urea and creatinine clearances), weekly Kt/V urea, 24-h urine output, and dialysate-to-plasma creatinine ratio at 4 h (D/P (creatinine) at 4 h). The medication history consisted of angiotensin-converting enzyme (ACE) inhibitors, angiotensin II receptor blockers (ARB), erythropoiesis stimulating agents (ESA), vitamin D, and calcium channel blockers. The baseline comorbid conditions, including diabetes mellitus, hypertension, hyperlipidemia and cardiovascular disease (CVD), were also collected for statistical analysis. Fasting blood samples were used to measure creatinine, albumin, total protein, glutamic-pyruvic transaminase (GPT), white blood cell (WBC) count, hemoglobin, ferritin, cholesterol, triglyceride, intact parathyroid hormone (PTH), calcium, and phosphate.

The study participants were classified as two groups by the median value of AGR (1.2) to evaluate the prognostic value of AGR on clinical outcomes: the high AGR group (≧1.2) and the low AGR group (< 1.2). Cardiovascular (CV) disease was the most common cause of patient mortality in our study cohort. This investigation was carried out to evaluate the impact of AGR on patient mortality from all causes, and CVD. Ethic approval was obtained from the institutional review board of Changhua Christian Hospital and the study was conducted in accordance with the ethical standards of the declaration of Helsinki. The individual written informed consent was exempted for a retrospective analysis of routine data in Taiwan.

### Statistical analysis

The distribution of categorical variables was expressed as frequencies and percentages. The continuous data were expressed as mean ± standard deviation (SD) or median (interquartile range, IQR) depending on whether the variable was normally distributed. Between-group differences were compared using the Student’s test or Mann–Whitney test for continuous variables while the categorical variables were compared by Chi-square test or Fisher’s exact test. The comparison of survival status for the two AGR groups was performed by the Kaplan–Meier method with log-rank test to determine to the significance. Cox proportional hazards model was used to evaluate the relationship between study covariates and study outcomes. The variables which contributed significantly (p < 0.05) to the outcome of interest in the univariate analysis were selected into the multivariate Cox regression analysis. The hazard ratio (HR) with 95% confident interval (CI) was used to show the significance. Potential candidate variables for multiple adjustments did not include albumin and total protein, because they are used in the calculation of AGR.

We performed five adjustment models to determine the prediction value of AGR in the risk for all-cause and CVD mortality. Model 1 was constructed by incorporating sex, age, smoking status, BMI, predialysis status, and the etiology of CKD; model 2 by adding medications use to those variables in model 1; model 3 by adding comorbidities to those variables in model 2; model 4 by adding PD-related measurements to model 3; model 5 by adding laboratory measurements to those variables in model 4. We also performed 4 sensitivity tests to strengthen our findings. First, the HR was calculated for every 1-unit decrease in AGR. Second, the optimal cut-off value of AGR for predicting mortality by receiver operating characteristic (ROC) curve analysis was used in the Cox models. Third, the Cox model was repeated with AGR treated as tertiles. Fourth, the HR was further adjusted by the propensity scores to mitigate the influence of unbalanced covarites distribtuion.

The association between AGR and baseline patient characteristics was evaluated by Pearson correlation test and multiple linear regression analysis. To compare the prediction capacity of study outcomes between AGR and other important clinical parameters, we conducted ROC analysis with area under the curve (AUC) analysis. The variables selected for AUC calculation included AGR, WBC counts, total protein, albumin, ferritin, and hemoglobin. Furthermore, the predictive values of those variables were also compared by the AUC calculated with each variable added to the variables in model 4. In addition, we also calculated the IDI to compare the discriminatory value for the predictive value of AGR in comparison to those laboratory variables. All the statistical analyses were performed using IBM SPSS Statistics for Windows, Version 22.0 (IBM Corp., Armonk, NY). A two-sided p value of < 0.05 was considered significant.

## References

[1] Deo R, Yang W, Khan AM (2014). Serum aldosterone and death, end-stage renal disease, and cardiovascular events in blacks and whites: Findings from the Chronic Renal Insufficiency Cohort (CRIC) Study. Hypertension.

[2] Liu KD, Yang W, Go AS (2015). Urine neutrophil gelatinase-associated lipocalin and risk of cardiovascular disease and death in CKD: Results from the Chronic Renal Insufficiency Cohort (CRIC) Study. Am. J. Kidney Dis..

[3] Tang WH, Wang Z, Kennedy DJ (2015). Gut microbiota-dependent trimethylamine N-oxide (TMAO) pathway contributes to both development of renal insufficiency and mortality risk in chronic kidney disease. Circ. Res..

[4] McMillan DC, Watson WS, O’Gorman P (2001). Albumin concentrations are primarily determined by the body cell mass and the systemic inflammatory response in cancer patients with weight loss. Nutr. Cancer..

[5] Gabay C, Kushner I (1999). Acute-phase proteins and other systemic responses to inflammation. N. Engl. J. Med..

[6] Adly L, Hill D, Sherman ME (2006). Serum concentrations of estrogens, sex hormone-binding globulin, and androgens and risk of breast cancer in postmenopausal women. Int. J. Cancer..

[7] Asher V, Lee J, Bali A (2012). Preoperative serum albumin is an independent prognostic predictor of survival in ovarian cancer. Med. Oncol..

[8] Guthrie GJ, Roxburgh CS, Farhan-Alanie OM (2013). Comparison of the prognostic value of longitudinal measurements of systemic inflammation in patients undergoing curative resection of colorectal cancer. Br. J. Cancer..

[9] Shibutani M, Maeda K, Nagahara H (2015). The pretreatment albumin to globulin ratio predicts chemotherapeutic outcomes in patients with unresectable metastatic colorectal cancer. BMC Cancer..

[10] Yao Y, Zhao M, Yuan D (2014). Elevated pretreatment serum globulin albumin ratio predicts poor prognosis for advanced non-small cell lung cancer patients. J. Thorac. Dis..

[11] Zhou T, He X, Fang W (2016). Pretreatment albumin/globulin ratio predicts the prognosis for small-cell lung cancer. Medicine.

[12] Pifer TB, McCullough KP, Port FK (2002). Mortality risk in hemodialysis patients and changes in nutritional indicators: DOPPS. Kidney Int..

[13] Lambie M, Chess J, Donovan KL (2013). Independent effects of systemic and peritoneal inflammation on peritoneal dialysis survival. J. Am. Soc. Nephrol..

[14] Nelson JJ, Liao D, Sharrett AR (2000). Serum albumin level as a predictor of incident coronary heart disease: The atherosclerosis Risk in Communities (ARIC) study. Am. J. Epidemiol..

[15] Danesh J, Collins R, Appleby P (1998). Association of fibrinogen, C-reactive protein, albumin, or leukocyte count with coronary heart disease: Meta-analyses of prospective studies. JAMA.

[16] Djoussé L, Rothman KJ, Cupples LA (2002). Serumalbumin and risk of myocardial infarction and all-cause mortalityin the Framingham offspring study. Circulation.

[17] Sabatino A, Regolisti G, Karupaiah T (2017). Protein-energy wasting and nutritional supplementation in patients with end-stage renal disease on hemodialysis. Clin. Nutr..

[18] Han SH, Han DS (2012). Nutrition in patients on peritoneal dialysis. Nat. Rev. Nephrol..

[19] Kopple JD (1997). McCollum Award Lecture, 1996: Protein-energy malnutrition in maintenance dialysis patients. Am. J. Clin. Nutr..

[20] Tayyem RF, Mrayyan MT (2008). Assessing the prevalence of malnutrition in chronic kidney disease patients in Jordan. J. Renal Nutr..

[21] Ikizler TA, Cano NJ, Franch H (2013). Prevention and treatment of protein energy wasting in chronic kidney disease patients: A consensus statement by the International Society of Renal Nutrition and Metabolism. Kidney Int..

[22] Kovesdy CP, Kopple JD, Kalantar-Zadeh K (2013). Management of protein-energy wasting in non-dialysis dependent chronic kidney disease reconciling low protein intake with nutritional therapy. Am. J. Clin. Nutr..

[23] Kwon YE, Kee YK, Yoon CY (2016). Change of nutritional status assessed using subjective global assessment is associated with all-cause mortality in incident dialysis patients. Medicine.

[24] Walsh PN, Mills DC, White JG (1977). Metabolism and function of human platelets washed by albumin density gradient separation. Br. J. Haematol..

[25] Ishikawa Y, Sasakawa S, Takase M (1984). Effect of albumin immobilization by plasma polymerization on platelet reactivity. Thromb. Res..

[26] Porcellati S, Gresele P, Stasi M (1995). Albumin prevents TxB, formation from thrombin-stimulated human platelets by sequestering the liberated arachidonic acid in the extracellular space. Platelets.

[27] Gresele P, Deckmyn H, Huybrechts E (1984). Serum albumin enhances the impairment of platelet aggregation with thromboxane synthase inhibition by increasing the formation of prostaglandin D2. Biochem. Pharmacol..

[28] Sloand EM, Bern MM, Kaldany A (1986). Effect on platelet function of hypoalbuminemia in peritoneal dialysis. Thromb. Res..

[29] Du XJ, Tang LL, Mao YP (2014). The pretreatment albumin to globulin ratio has predictive value for long term mortality in nasopharyngeal carcinoma. PLoS ONE.

[30] Akchurin OM, Kaskel F (2015). Update on inflammation in chronic kidney disease. Blood Purif..

[31] Stenvinkel P (2013). Can treating persistent inflammation limit protein energy wasting?. Semin. Dial.

[32] Azab BN, Bhatt VR, Vonfrolio S (2013). Value of the pretreatment albumin to globulin ratio in predicting long-term mortality in breast cancer patients. Am. J. Surg..

[33] Beamer N, Coull BM, Sexton G (1993). Fibrinogen and the albumin–globulin ratio in recurrent stroke. Stroke.

[34] Azab B, Bibawy J, Harris K (2013). Value of albumin–globulin ratio as a predictor of all-cause mortality after non-ST elevation myocardial infarction. Angiology.

